# Association between obstructive sleep apnea and the risk of cerebrovascular disease in COPD patients

**DOI:** 10.3389/fmed.2025.1742688

**Published:** 2026-01-09

**Authors:** Jie Quan, Qiujing Tang, Deyi Zhou, Yihuan Su, Nuoyan Huang, Yating Liu, Yutong Lu, Boyang Xiao, Zhenzhen Zheng, Yingmei Luo, Weimin Yao

**Affiliations:** 1 The Second Affiliated Hospital of Guangdong Medical University, Zhanjiang, Guangdong, China; 2 School of Basic Medical Sciences, Guangdong Medical University, Zhanjiang, Guangdong, China

**Keywords:** cerebrovascular disease, chronic obstructive pulmonary disease, diagnosed, obstructive sleep apnea, risk factors

## Abstract

**Purpose:**

This study aimed to investigate the association between obstructive sleep apnea (OSA) and the risk of cerebrovascular disease in patients with chronic obstructive pulmonary disease (COPD), as well as to identify potential influencing factors.

**Methods:**

This retrospective cohort study enrolled 1,189 patients with chronic obstructive pulmonary disease (COPD) diagnosed at the Second Affiliated Hospital of Guangdong Medical University from January 2016 to January 2020. Among these patients, 1,057 had no obstructive sleep apnea (OSA), whereas 132 were diagnosed with OSA (45 mild, 52 moderate, and 35 severe). Data were obtained from the hospital’s electronic medical record system, and patients were followed up until August 2025, or until they developed cerebrovascular disease, were lost to follow-up, or died. Univariate and multivariate logistic regression analyses were performed to evaluate the association between OSA severity and the risk of cerebrovascular disease, and sex-stratified analyses were also conducted.

**Results:**

Multivariate analysis demonstrated that the severity of obstructive sleep apnea (OSA) was significantly and positively associated with the risk of cerebrovascular disease among patients with chronic obstructive pulmonary disease (COPD). The adjusted odds ratios (ORs) for cerebrovascular disease in patients with mild, moderate, and severe OSA were 2.19 (95% CI: 1.08–4.42, *p* = 0.029), 2.79 (95% CI: 1.47–5.28, *p* = 0.002), and 3.78 (95% CI: 1.62–8.81, *p* = 0.002), respectively. Additionally, smoking history (OR = 4.14, 95% CI: 3.07–5.58, *p* < 0.001), hyperlipidemia (OR = 1.83, 95% CI: 1.30–2.56, *p* < 0.001), and hypertension (OR = 2.92, 95% CI: 2.16–3.96, *p* < 0.001) were identified as independent predictors of cerebrovascular disease in COPD patients. Sex-stratified analysis revealed distinct risk profiles between male and female patients, with OSA exerting a more pronounced effect on cerebrovascular disease risk among males. Age-stratified analysis further showed that among patients aged ≥70 years, OSA had a stronger association with cerebrovascular disease risk compared with younger patients.

**Conclusion:**

The severity of obstructive sleep apnea (OSA) was positively and proportionally associated with the risk of cerebrovascular disease among patients with chronic obstructive pulmonary disease (COPD). The association between OSA and cerebrovascular disease was more pronounced among male patients and individuals aged ≥70 years. Strengthened preventive and management strategies for cerebrovascular disease should be prioritized in COPD patients, especially those with concomitant OSA.

## Background

Obstructive sleep apnea (OSA) is a prevalent sleep-related breathing disorder characterized by repeated episodes of apnea and hypopnea during sleep, resulting in intermittent hypoxemia and fragmented sleep architecture (1). Chronic obstructive pulmonary disease (COPD) is a progressive inflammatory airway disorder characterized by persistent airflow limitation, typically presenting with chronic cough, sputum production, and exertional dyspnea (2). In recent years, a substantial body of evidence has indicated a significant association between obstructive sleep apnea (OSA) and chronic obstructive pulmonary disease (COPD), and this overlap syndrome may further increase the risk of cerebrovascular disease in affected individuals (3–5). The shared pathophysiological mechanisms of obstructive sleep apnea (OSA) and chronic obstructive pulmonary disease (COPD) can be summarized as follows: First, both disorders are characterized by airway inflammation. In OSA, upper airway inflammation contributes to pharyngeal narrowing and collapse, whereas in COPD, lower airway inflammation results in persistent airflow obstruction. Second, both diseases are strongly associated with increased oxidative stress. Recurrent nocturnal hypoxemia and hypercapnia in OSA lead to elevated systemic oxidative stress, whereas COPD-related oxidative stress primarily arises from chronic exposure to noxious particles or gasses—most notably tobacco smoke—which in turn damages pulmonary tissue. Moreover, individuals with OSA and COPD frequently exhibit systemic inflammation, which may further increase the risk of comorbid cardiovascular diseases. Finally, both OSA and COPD may lead to the development of pulmonary hypertension, driven by nocturnal hypoxemia and pulmonary vasoconstriction, which further increase cardiopulmonary load and impair overall physiological function (6, 7).

Cerebrovascular disease (CVD) remains a major global cause of mortality and long-term disability. Its pathogenesis is multifactorial, involving a constellation of risk factors such as hypertension, hyperlipidemia, diabetes mellitus, and cigarette smoking (8). Recent evidence has established obstructive sleep apnea (OSA) as an independent and significant risk factor for cerebrovascular disease (CVD). In individuals with OSA, recurrent nocturnal hypoxemia and sympathetic overactivation contribute to vascular endothelial dysfunction, systemic inflammation, and hemorheological disturbances, thereby exacerbating cerebrovascular risk (9).

The coexistence of obstructive sleep apnea (OSA) and chronic obstructive pulmonary disease (COPD), often referred to as the “overlap syndrome,” is relatively prevalent among COPD patients (10). Patients with chronic obstructive pulmonary disease (COPD) inherently exhibit airflow limitation and chronic hypoxemia; when coexisting with obstructive sleep apnea (OSA), nocturnal oxygen desaturation is further aggravated, leading to more pronounced hemodynamic instability and systemic inflammation, thereby heightening the risk of cerebrovascular events (4). However, existing evidence regarding the association between obstructive sleep apnea (OSA) and cerebrovascular disease (CVD) in patients with chronic obstructive pulmonary disease (COPD) remains limited and inconclusive. Therefore, the present study aimed to elucidate the association between OSA and CVD in COPD patients through a retrospective cohort design and to identify potential contributing factors.

## Methods

### Study design

This study adopted a retrospective cohort design to examine the association between obstructive sleep apnea (OSA) and cerebrovascular disease (CVD) among patients with chronic obstructive pulmonary disease (COPD). The study population comprised patients diagnosed with COPD between January 2016 and January 2020 at the Second Affiliated Hospital of Guangdong Medical University, with no prior history of cerebrovascular disease. The diagnosis of OSA was ascertained from discharge records documented in the hospital’s electronic medical record system, using Philips A5/A6 polysomnography (PSG) equipment. Participants were followed from baseline until August 2025, or until the first occurrence of CVD diagnosis, loss to follow-up, or death. The primary endpoint was defined as the first occurrence of newly diagnosed CVD during the follow-up period.

### Sources of data

Data were collected through a review of the hospital’s electronic medical record (EMR) system for the period January 2016 to January 2020. Follow-up was primarily conducted through the hospital’s electronic medical record (EMR) system, which was reviewed to verify whether patients had attended outpatient, emergency, or inpatient services at the study center. Particular attention was given to documenting any head computed tomography (CT) or related neuroimaging evaluations. For patients who did not return for scheduled follow-up visits, telephone interviews were conducted to determine their health status and to confirm whether cerebrovascular disease (CVD) had been diagnosed at external medical institutions. Key clinical information, including diagnosis dates and the names of medical institutions, was collected for verification. The clinical data of the subjects include general information: age, gender, smoking history, comorbidities (diabetes, hypertension, hyperlipidemia, coronary heart disease), laboratory indicators (partial pressure of carbon dioxide, partial pressure of oxygen, white blood cells, aspartate aminotransferase, uric acid). This study was conducted in accordance with the principles of the Declaration of Helsinki and received ethical approval from the Medical Ethics Committee of the Second Affiliated Hospital of Guangdong Medical University (Approval No. YJKT202505001). Written informed consent was obtained from all participants or their legal guardians prior to study enrollment.

Inclusion criteria: ① aged ≥18 years; ② who fulfilled the diagnostic criteria for chronic obstructive pulmonary disease (COPD). The diagnosis was established based on the presence of persistent airflow limitation, confirmed by spirometric assessment of pulmonary function (2). The core diagnostic criterion was a post-bronchodilator forced expiratory volume in 1 s to forced vital capacity ratio (FEV₁/FVC) < 0.70, confirming persistent airflow limitation. Exclusion criterion: ① Patients with incomplete clinical information or missing key variables; ② Those who did not sign the informed consent form.

The diagnostic classification of obstructive sleep apnea (OSA) was based on the apnea–hypopnea index (AHI) as follows: AHI < 5 events/h was considered normal (adults); 5–14.9 events/h indicated mild OSA; 15–29.9 events/h, moderate OSA; and ≥30 events/h, severe OSA (11). Cerebrovascular disease (CVD) encompasses a spectrum of disorders affecting the cerebral vasculature and cerebral circulation, including conditions that cause acute disruption of cerebral blood flow and subsequent neuronal injury, as well as those that induce chronic small-vessel pathology and progressive neurological dysfunction (8). In this study, incident cerebrovascular disease (CVD) was defined to include ischemic stroke, hemorrhagic stroke, transient ischemic attack (TIA), and subarachnoid hemorrhage (SAH).

### Statistical analysis

All statistical analyses were conducted using SPSS version 25.0 (IBM Corp., Armonk, NY, United States) and RStudio (R Foundation for Statistical Computing, Vienna, Austria), with statistical significance defined as a two-sided *p*-value < 0.05. Continuous variables were expressed as mean ± standard deviation (SD), and categorical variables were summarized as frequencies and percentages. Comparisons of categorical variables were performed using *χ*^2^ tests or Fisher’s exact tests, while continuous variables were compared using Student’s *t*-tests or Wilcoxon rank-sum tests, as appropriate. Univariate and multivariate logistic regression analyses were conducted to evaluate the association between potential risk factors and cerebrovascular disease (CVD), yielding odds ratios (ORs) with 95% confidence intervals (CIs) after adjustment for confounders. Additionally, conduct gender and age stratified analysis to further explore the differences in risk of cerebrovascular diseases among patients of different genders and ages.

## Results

### Baseline characteristics

A total of 1,189 patients with chronic obstructive pulmonary disease (COPD) were included in the study, of whom 1,057 had no obstructive sleep apnea (OSA) and 132 were diagnosed with OSA (45 mild, 52 moderate, and 35 severe cases). Baseline characteristics of the study population are summarized in Table 1. Significant differences were observed among groups in terms of age, sex, smoking history, and comorbid conditions. For example, the proportion of male patients was higher in the OSA group (*p* = 0.050); smoking history was more frequent (*p* < 0.001); and the prevalence of hypertension and hyperlipidemia was significantly greater (both *p* < 0.001) among patients with OSA.

**Table 1:** Baseline characteristics.

Variable	Overall (*n* = 1,189)	Non-OSA (*n* = 1,057)	Mild OSA (*n* = 45)	Moderate OSA (*n* = 52)	Severe OSA (*n* = 35)	*P*
Age (years)	77.34 ± 9.93	77.18 ± 10.03	79.20 ± 8.98	77.29 ± 9.78	79.97 ± 7.83	0.230
Gender						0.050
Male	887 (74.60%)	776 (73.42%)	36 (80.00%)	44 (84.62%)	31 (88.57%)	
Female	302 (25.40%)	281 (26.58%)	9 (20.00%)	8 (15.38%)	4 (11.43%)	
Smoking						<0.001
No	852 (71.66%)	776 (73.42%)	23 (51.11%)	33 (63.46)	20 (57.14%)	
Yes	337 (28.34%)	281 (26.58%)	22 (48.89%)	19 (36.54%)	15 (42.86)	
Partial Pressure of Carbon Dioxide (mmHg)	42.37 ± 13.32	42.34 ± 13.12	46.83 ± 19.73	42.09 ± 10.36	38.10 ± 11.83	0.034
Partial pressure of oxygen (mmHg)	104.47 ± 35.37	103.79 ± 35.11	109.69 ± 33.85	108.87 ± 38.75	111.74 ± 39.49	0.302
White blood cell count (10^9^/L)	9.69 ± 5.58	9.80 ± 5.72	9.53 ± 5.48	8.22 ± 2.84	8.89 ± 4.16	0.192
Alanine aminotransferase (U/L)	26.40 ± 80.81	26.72 ± 84.92	27.15 ± 32.89	17.66 ± 13.39	28.72 ± 49.23	0.883
Aspartate aminotransferase (U/L)	27.59 ± 28.09	27.26 ± 27.45	29.52 ± 27.33	27.36 ± 24.15	35.45 ± 47.40	0.377
Uric acid (umol/L)	341.81 ± 128.71	340.48 ± 128.59	352.16 ± 155.74	356.73 ± 131.72	346.43 ± 85.12	0.766
Hyperlipidemia						0.003
No	931 (78.30%)	832 (78.71%)	39 (86.67%)	41 (78.85%)	19 (54.29%)	
Yes	258 (21.70%)	225 (21.29%)	6 (13.33%)	11 (21.15%)	16 (45.71%)	
Diabetes						<0.001
No	1,083 (91.08%)	972 (91.96%)	36 (80.00%)	48 (92.31%)	27 (77.14%)	
Yes	106 (8.92%)	85 (8.04%)	9 (20.00%)	4 (7.69%)	8 (22.86%)	
Hypertension						<0.001
No	778 (65.43%)	709 (67.08%)	24 (53.33%)	32 (61.54%)	13 (37.14%)	
Yes	411 (34.57%)	348 (32.92%)	21 (46.67%)	20 (38.46%)	22 (62.86%)	
Coronary heart disease						<0.001
No	995 (83.68%)	902 (85.34%)	33 (73.33%)	43 (82.69%)	17 (48.57%)	
Yes	194 (16.32%)	155 (14.66%)	12 (26.67%)	9 (17.31%)	18 (51.43%)	

### Cerebrovascular disease risk analysis

**Figure 1:**
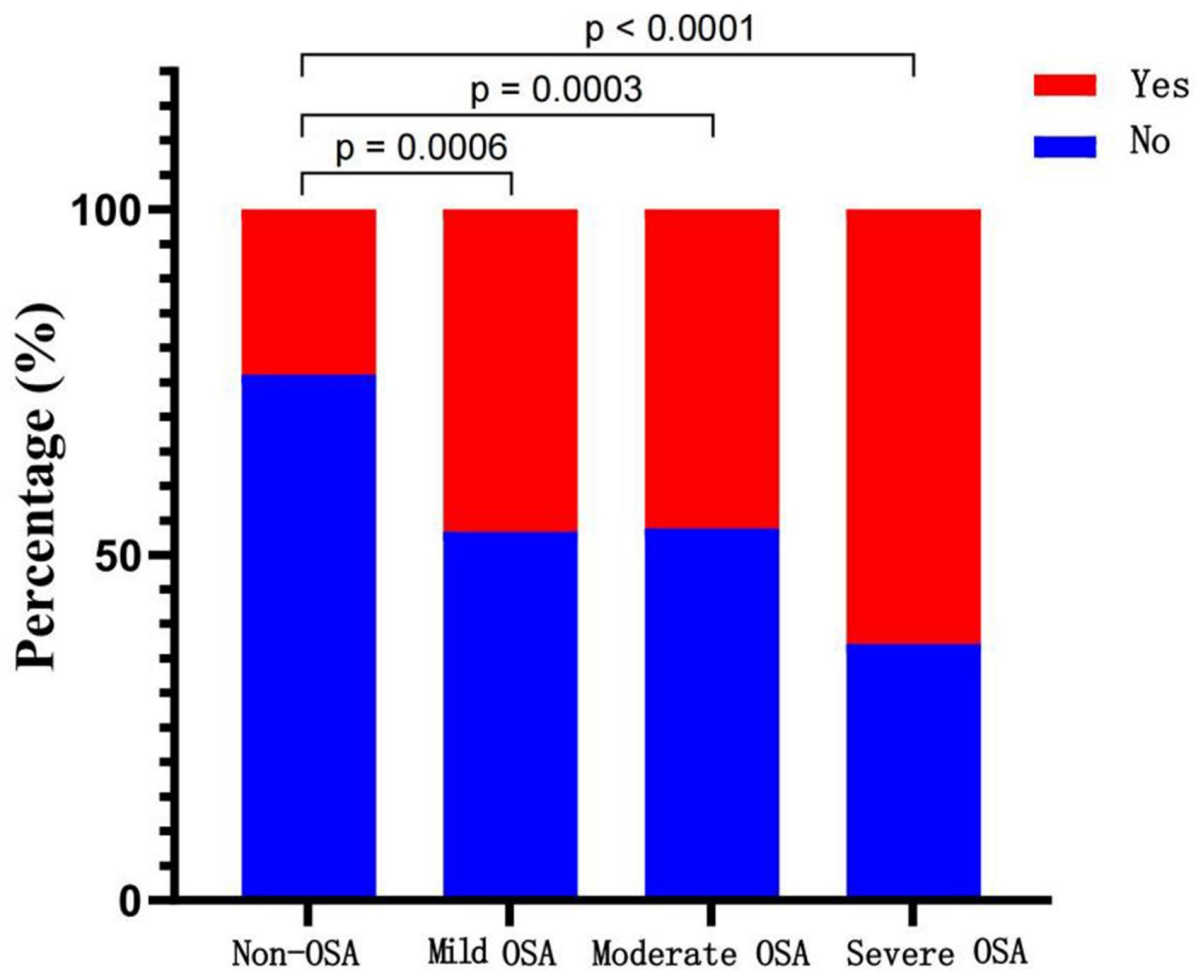
Probability of cerebrovascular disease by OSA severity.

Table 2 summarizes the results of the cerebrovascular disease (CVD) risk analysis for all participants. Univariate analysis revealed that gender, smoking history, hyperlipidemia, diabetes mellitus, hypertension, coronary heart disease (CHD), and cardiovascular disease (CVD) were all significantly associated with the occurrence of CVD. After adjusting for these confounders in the multivariate logistic regression model, gender (OR = 1.66, 95% CI: 1.20–2.28, *p* = 0.002), smoking history (OR = 4.14, 95% CI: 3.07–5.58, *p* < 0.001), hyperlipidemia (OR = 1.83, 95% CI: 1.30–2.56, *p* < 0.001), and hypertension (OR = 2.92, 95% CI: 2.16–3.96, *p* < 0.001) remained independently associated with CVD risk. Furthermore, the severity of obstructive sleep apnea (OSA) showed a positive correlation with the risk of developing CVD. Patients with mild (OR = 2.19, 95% CI: 1.08–4.42, *p* = 0.029), moderate (OR = 2.79, 95% CI: 1.47–5.28, *p* = 0.002), and severe OSA (OR = 3.78, 95% CI: 1.62–8.81, *p* = 0.002) all exhibited a significantly increased risk of CVD. A progressive increase in OSA severity was associated with a higher incidence of CVD (Figure 1). The probabilities of CVD occurrence among the non-OSA, mild OSA, moderate OSA, and severe OSA groups were 0.239, 0.467, 0.462, and 0.629, respectively. All OSA subgroups demonstrated statistically significant differences (*p* < 0.05) compared with the non-OSA group.

**Table 2:** Cerebrovascular disease incidence among all study participants.

Variable	Univariate analysis		Multivariable analysis			
	OR	95%CI	*P*	OR	95%CI	*P*
Gender	1.53	(1.15,2.03)	0.003	1.66	(1.20,2.28)	0.002
Smoking	4.229	(3.21,5.57)	<0.001	4.14	(3.07,5.58)	<0.001
Hyperlipidemia	2.50	(1.87,3.34)	<0.001	1.83	(1.30,2.56)	<0.001
Diabetes	1.74	(1.15,2.63)	0.009	0.87	(0.54,1.42)	0.581
Hypertension	3.42	(2.62,4.47)	<0.001	2.92	(2.16,3.96)	<0.001
Coronary Heart Disease	2.09	(1.52,2.89)	<0.001	1.10	(0.73,1.64)	0.655
PaCO₂ (mmHg)	0.99	(0.98,1.00)	0.166			
Non-OSA	1.00			1.00		
Mild OSA	2.78	(1.52,5.08)	<0.001	2.19	(1.08,4.42)	0.029
Moderate OSA	2.72	(1.55,4.78)	<0.001	2.79	(1.47,5.28)	0.002
Severe OSA	5.38	(2.67,10.83)	<0.001	3.78	(1.62,8.81)	0.002

### Sex-stratified analysis

Sex-stratified analysis (Table 3) demonstrated that, among male patients, smoking history (OR = 3.39, 95% CI: 2.38–4.81, *p* < 0.001), hyperlipidemia (OR = 2.04, 95% CI: 1.37–3.03, *p* < 0.001), and hypertension (OR = 2.73, 95% CI: 1.90–3.93, *p* < 0.001) were significantly correlated with the occurrence of cerebrovascular disease (CVD). Mild (OR = 2.88, 95% CI: 1.32–6.28, *p* = 0.008), moderate (OR = 2.97, 95% CI: 1.50–5.86, *p* = 0.002), and severe OSA (OR = 4.16, 95% CI: 1.72–10.04, *p* = 0.002) were all significantly associated with an elevated risk of CVD among male patients. Among female patients (Table 4), smoking history (OR = 7.60, 95% CI: 4.14–13.96, *p* < 0.001) and hypertension (OR = 3.88, 95% CI: 2.16–6.98, p < 0.001) were significantly associated with CVD, whereas hyperlipidemia (OR = 1.46, 95% CI: 0.75–2.83, *p* = 0.269) did not reach statistical significance. Although severe OSA (OR = 6.37, 95% CI: 0.85–62.06, *p* = 0.111) appeared to confer a higher risk among female patients, the association was not statistically significant—likely due to the limited sample size and small number of cerebrovascular events, which resulted in a wide confidence interval crossing unity and consequently a non-significant *p*-value.

**Table 3:** Risk of cerebrovascular disease in males.

Variable	Univariate analysis			Multivariable analysis		
	OR	95%CI	*P*	OR	95%CI	*P*
Smoking	3.77	(2.73,5.22)	<0.001	3.39	(2.38,4.81)	<0.001
Hyperlipidemia	2.63	(1.87,3.70)	<0.001	2.04	(1.37,3.03)	<0.001
Diabetes	1.74	(1.05,2.88)	0.031	0.88	(0.49,1.57)	0.667
Hypertension	3.49	(2.54,4.79)	<0.001	2.73	(1.90,3.93)	<0.001
CHD	2.36	(1.61,3.46)	<0.001	1.18	(0.73,1.91)	0.503
PaCO₂ (mmHg)	0.99	(0.98,1.00)	0.262			
No OSA	1.00			1.00		
Mild OSA	3,76	(1.91,7.39)	<0.001	2.88	(1.32,6.28)	0.008
Moderate OSA	2.86	(1.54,5.32)	<0.001	2.97	(1.50,5.86)	0.002
Severe OSA	5.95	(2.83,12.52)	<0.001	4.16	(1.72,10.04)	0.002

**Table 4:** Risk of cerebrovascular disease in females.

Variable	Univariate analysis			Multivariable analysis		
	OR	95%CI	*P*	OR	95%CI	*P*
Smoking	5.87	(3.43,10.06)	<0.001	7.60	(4.14,13.96)	<0.001
Hyperlipidemia	2.26	(1.28,3.96)	0.005	1.46	(0.75,2.83)	0.269
Diabetes	1.64	(0.78,3.44)	0.194			
Hypertension	3.08	(1.88,5.05)	<0.001	3.88	(2.16,6.98)	<0.001
CHD	1.50	(0.83,2.72)	0.182			
PaCO₂ (mmHg)	0.99	(0.98,1.01)	0.422			
No OSA	1.00			1.00		
Mild OSA	1.06	(0.26,4.34)	0.934			
Moderate OSA	3.54	(0.83,15.13)	0.088			
Severe OSA	6.37	(0.85,62.06)	0.111			

### Age-stratified analysis

Age-stratified analysis revealed distinct risk profiles across age groups. Among patients aged ≥70 years (Table 5), male sex (OR = 1.61, 95% CI: 1.14–2.27, *p* = 0.007), smoking history (OR = 3.92, 95% CI: 2.83–5.43, *p* < 0.001), hyperlipidemia (OR = 1.77, 95% CI: 1.23–2.55, *p* = 0.002), and hypertension (OR = 2.59, 95% CI: 1.87–3.58, *p* < 0.001) were significantly associated with cerebrovascular disease (CVD). Furthermore, all severity levels of obstructive sleep apnea (OSA) were significantly associated with an increased risk of CVD in this age group: mild OSA (OR = 2.04, 95% CI: 1.01–4.14, *p* = 0.047), moderate OSA (OR = 3.06, 95% CI: 1.50–6.24, *p* = 0.002), and severe OSA (OR = 3.75, 95% CI: 1.49–9.42, *p* = 0.005). In contrast, among patients aged <70 years (Table 6), smoking history (OR = 4.68, 95% CI: 2.11–10.40, *p* < 0.001) and hypertension (OR = 4.93, 95% CI: 2.13–11.40, *p* < 0.001) remained significant predictors of CVD. However, the presence of OSA was not significantly associated with an increased risk of CVD in this younger age group.

**Table 5:** Cerebrovascular disease risk among participants aged 70 years or older.

Variable	Univariate analysis			Multivariable analysis		
	OR	95%CI	*P*	OR	95%CI	*P*
Gender	1.47	(1.08,2.00)	0.014	1.61	(1.14,2.27)	0.007
Smoking	4.08	(3.02,5.52)	<0.001	3.92	(2.83,5.43)	<0.001
Hyperlipidemia	2.28	(1.66,3.13)	<0.001	1.77	(1.23,2.55)	0.002
Diabetes	1.90	(1.20,2.99)	0.006	1.03	(0.61,1.74)	0.915
Hypertension	2.89	(2.17,3.86)	<0.001	2.59	(1.87,3.58)	<0.001
CHD	1.79	(1.28,2.51)	<0.001	1.00	(0.66,1.53)	0.991
Cardiovascular disease	1.74	(1.30,2.31)	<0.001	1.24	(0.88,1.75)	0.223
Glucocorticoid use	0.72	(0.50,1.04)	0.082			
PaCO₂ (mmHg)	0.99	(0.98,1.00)	0.061			
No OSA	1.00			1.00		
Mild OSA	2.57	(1.39,4.77)	0.003	2.04	(1.01,4.14)	0.047
Moderate OSA	3.12	(1.66,5.87)	<0.001	3.06	(1.50,6.24)	0.002
Severe OSA	6.28	(2.83,13.92)	<0.001	3.75	(1.49,9.42)	0.005

**Table 6:** Cerebrovascular disease risk among participants under 70 years.

Variable	Univariate analysis			Multivariable analysis		
	OR	95%CI	*P*	OR	95%CI	*P*
Gender	1.73	(0.79,3.78)	0.172			
Smoking	5.46	(2.59,11.50)	<0.001	4.68	(2.11,10.40)	<0.001
Hyperlipidemia	3.37	(1.52,7.48)	0.003	1.83	(0.71,4.70)	0.209
Diabetes	1.01	(0.28,3.61)	0.986			
Hypertension	6.69	(3.14,14.26)	<0.001	4.93	(2.13,11.40)	<0.001
CHD	2.94	(0.97,8.94)	0.057			
PaCO₂ (mmHg)	1.02	(0.99,1.05)	0.276			
No OSA	1.00			1.00		
Mild OSA	0.00	(0.00,0.00)	0.999			
Moderate OSA	1.45	(0.30,7.02)	0.643			
Severe OSA	1.63	(0.18,15.07)	0.665			

## Discussion

This study is the first to confirm the relationship between OSA severity and CVD risk in COPD patients, and analyzes the differences in CVD risk by age and gender. This retrospective cohort study examined the association between obstructive sleep apnea (OSA) and cerebrovascular disease (CVD) in patients with chronic obstructive pulmonary disease (COPD). The findings demonstrated a significant positive association between OSA severity and the risk of developing CVD among patients with COPD. Moreover, smoking history, hyperlipidemia, and hypertension were identified as independent predictors of CVD in patients with COPD. Stratified analyses further revealed sex- and age-specific differences in CVD risk, with OSA exerting a more pronounced effect among male patients and individuals aged ≥70 years.

### Relationship between OSA and cerebrovascular disease

This study demonstrated a significant positive association between OSA severity and the risk of cerebrovascular disease (CVD) in patients with chronic obstructive pulmonary disease (COPD). This finding aligns with prior research. In patients with OSA, recurrent nocturnal apnea and hypopnea induce intermittent hypoxemia and sympathetic nervous system activation, which in turn cause vascular endothelial dysfunction, systemic inflammation, and hemorheological disturbances. These pathophysiological alterations may contribute to an elevated risk of CVD (12). Moreover, patients with OSA often have comorbidities such as hypertension and hyperlipidemia, which may act synergistically to further increase the risk of cerebrovascular disease (CVD) (13).

In this study, the odds ratios (ORs) for cerebrovascular disease (CVD) risk in patients with mild, moderate, and severe OSA were 2.19, 2.79, and 3.78, respectively, all of which were statistically significant. This suggests that as the severity of OSA increases, the risk of developing CVD in COPD patients rises correspondingly. Thus, in COPD patients with comorbid OSA, active management of OSA should be prioritized to mitigate the risk of CVD. OSA treatment measures include lifestyle modifications, such as weight loss, alcohol abstinence, and sleep posture adjustments, in addition to devices like continuous positive airway pressure (CPAP) (14, 15). These interventions not only improve patients’ sleep quality and daytime functionality but also decrease the incidence of cardiovascular events (16). Moreover, studies indicate that the association between obstructive sleep apnea (OSA) and cerebrovascular disease (CVD) may involve additional mechanisms, including oxidative stress and autonomic nervous system dysfunction (17, 18). These mechanisms may induce structural and functional changes in the vascular wall, thereby enhancing susceptibility to cerebrovascular disease (CVD). Therefore, future research should continue to explore the underlying mechanisms linking OSA to CVD and identify more effective intervention strategies.

### Gender differences

Sex-stratified analysis revealed distinct differences in cerebrovascular disease (CVD) risk factors between male and female patients. Among male patients, smoking history, hyperlipidemia, and hypertension were significantly associated with CVD, and the impact of obstructive sleep apnea (OSA) on CVD was more pronounced. In female patients, smoking history and hypertension were significantly associated with CVD; however, the effect of OSA did not reach statistical significance. This discrepancy may be attributed to gender-specific differences in pathophysiological mechanisms. Male patients may exhibit greater susceptibility to hypoxemia induced by OSA, whereas female patients may be influenced by other factors, such as hormonal fluctuations. For example, estrogen may exert a protective effect in females, potentially mitigating the impact of OSA on cerebrovascular health (19). Moreover, female patients may be more susceptible to social and psychological factors, which could further influence their risk of cerebrovascular disease (CVD) (20). Therefore, in clinical practice, individualized cerebrovascular disease (CVD) prevention strategies should be tailored to gender-specific characteristics. For male patients, particular attention should be given to OSA screening and treatment, in addition to the management of smoking and hypertension. For female patients, greater emphasis should be placed on monitoring hormonal fluctuations and implementing interventions targeting social and psychological factors. Through these personalized prevention strategies, the risk of CVD can be more effectively mitigated.

### Age differences

Age-stratified analysis further revealed distinct risk factors for cerebrovascular disease (CVD) among different age groups. In patients aged ≥70 years, male sex, smoking history, hyperlipidemia, and hypertension were significantly associated with the occurrence of CVD. Mild, moderate, and severe OSA were all significantly associated with an increased risk of CVD in patients aged ≥70 years. In patients aged <70 years, smoking history and hypertension were significantly associated with CVD; however, the presence of OSA did not significantly increase CVD risk. These results suggest that the impact of OSA on CVD is more pronounced in patients aged ≥70 years. This may be attributed to the physiological and pathological characteristics of older adults. Elderly patients exhibit reduced vascular wall elasticity and decreased tolerance to hypoxemia, increasing their susceptibility to vascular endothelial dysfunction and systemic inflammation (21). Moreover, older adults frequently have multiple chronic comorbidities, such as hypertension and hyperlipidemia, and the synergistic effects of these conditions with obstructive sleep apnea (OSA) may further elevate the risk of cerebrovascular disease (CVD) (22). Therefore, in clinical practice, particular emphasis should be placed on OSA screening and treatment for COPD patients aged ≥70 years, alongside the management of comorbid chronic conditions. For patients aged <70 years, although OSA does not significantly impact cerebrovascular disease risk, it remains crucial to address other risk factors, such as smoking and hypertension, to mitigate the risk of cerebrovascular disease (CVD) (23).

### Risk factors

This study further identified smoking, hypertension, and hyperlipidemia as independent risk factors for cerebrovascular disease (CVD) in patients with chronic obstructive pulmonary disease (COPD), consistent with existing literature. Both smoking and hypertension contribute to vascular endothelial dysfunction, systemic inflammation, and hemorheological disturbances, thereby elevating the risk of CVD (24). Therefore, for COPD patients, especially those with comorbid obstructive sleep apnea (OSA), smoking cessation should be actively encouraged to reduce the risk of cerebrovascular disease (CVD). Smoking cessation not only improves pulmonary function and quality of life but also decreases the incidence of cardiovascular events (25, 26).

### Clinical implications and future research directions

The findings of this study demonstrate a significant positive association between OSA severity and the risk of cerebrovascular disease (CVD) in patients with chronic obstructive pulmonary disease (COPD). Therefore, for COPD patients with comorbid OSA, active management of OSA should be prioritized to mitigate the risk of CVD. Moreover, smoking history, hyperlipidemia, and hypertension are independent predictors of CVD in COPD patients and should be carefully addressed through intervention strategies. Future research should focus on elucidating the specific mechanisms underlying the association between OSA and CVD in COPD patients. Prospective cohort studies and randomized controlled trials should be conducted to validate these findings and provide more robust evidence to guide clinical interventions.

Although this study provides valuable insights into the risk of cerebrovascular disease (CVD) in COPD patients with obstructive sleep apnea (OSA), several limitations must be acknowledged. First, as a retrospective cohort study, it is susceptible to both selection bias and information bias. The reliance on pre-existing medical records may introduce inconsistencies in data documentation across different clinicians and time periods. Furthermore, the absence of randomized allocation means that unmeasured confounding variables could influence the observed associations between OSA severity and CVD risk in COPD patients. Second, although multiple confounding factors were accounted for in the statistical analysis, unidentified or unmeasured confounders may still influence the results. For instance, lifestyle factors, such as dietary habits and physical activity levels, may be associated with CVD but were not fully considered in this study. Furthermore, in this study, only COPD patients suspected of having OSA underwent sleep monitoring, which may have excluded patients with mild OSA symptoms. Future research should employ prospective designs, extend follow-up periods, and more comprehensively collect and analyze potential confounding factors to validate these findings.

## Conclusion

The findings of this study demonstrate a significant positive association between the severity of obstructive sleep apnea (OSA) and the risk of cerebrovascular disease (CVD) in patients with chronic obstructive pulmonary disease (COPD). Factors such as smoking history, hyperlipidemia, and hypertension were identified as independent predictors of CVD in this population. The impact of OSA on CVD was more pronounced in male patients and those aged ≥70 years. These findings underscore the importance of intensified prevention and management of CVD in COPD patients, particularly those with comorbid OSA.

## Data Availability

The datasets presented in this study can be found in online repositories. The names of the repository/repositories and accession number(s) can be found in the article/Supplementary material.
